# CIDE-A is expressed in liver of old mice and in type 2 diabetic mouse liver exhibiting steatosis

**DOI:** 10.1186/1476-5926-6-4

**Published:** 2007-05-01

**Authors:** Bruce Kelder, Keith Boyce, Andres Kriete, Ryan Clark, Darlene E Berryman, Sheila Nagatomi, Edward O List, Mark Braughler, John J Kopchick

**Affiliations:** 1 Edison Biotechnology Institute, Ohio University, Athens, OH 45701, USA; 2 Clinical Data Inc, Newton, MA 02458, USA; 3 Department of Biomedical Sciences, College of Osteopathic Medicine, Ohio University, Athens, OH 45701, USA; 4 Immune Tolerance Network, Pittsburgh, PA 15238, USA; 5 Drexel University and Coriell Bioinformatics Initiative, School of Biomedical Engineering, Drexel University, Philadelphia, PA 19104, USA; 6 School of Human and Consumer Sciences, Ohio University, Athens, OH 45701, USA; 7 Rheogene, Norristown, PA 19403, USA

## Abstract

**Background:**

Increased levels of circulating fatty acids caused by insulin resistance and increased adipocyte lipolysis can accumulate within the liver resulting in steatosis. This steatosis sensitizes the liver to inflammation and further injury which can lead to liver dysfunction. We performed microarray analysis on normal mouse liver tissue at different ages and type 2 diabetic liver exhibiting steatosis to identify differentially expressed genes involved in lipid accumulation and liver dysfunction.

**Results:**

Microarray analysis identified CIDE-A as the most differentially expressed gene as a function of age. Mice fed a high fat diet developed hyperinsulinemia, hyperglycemia and liver steatosis, all features of the human metabolic syndrome. Increased CIDE-A expression was observed in type 2 diabetic liver and the elevated CIDE-A expression could be reversed by weight loss and normalization of plasma insulin. Also, CIDE-A expression was found to be correlated with hepatic lipid accumulation.

**Conclusion:**

The corresponding increase in CIDE-A expression with hyperinsulinemia and liver steatosis suggests a novel pathway for lipid accumulation in the liver.

## Background

Non-alcoholic fatty liver disease (NAFLD) is one of the most common causes of liver disease and is estimated to affect 10 to 24% of the general population in western nations [1]. While NAFLD is a serious problem, effective treatments are still lacking. NAFLD is characterized by a wide spectrum of liver damage ranging from simple steatosis to steatohepatitis (NASH) to advanced fibrosis and cirrhosis [2]. Hepatic steatosis is caused by lipid accumulation within hepatocytes and is a relatively benign condition. However, steatosis combined with necro-inflammatory activity may progress to end-stage liver disease [3-7]. The higher prevalence of NAFLD in persons with obesity, hyperinsulinemia or type 2 diabetes suggests that elevated circulating fatty acid concentrations caused by insulin resistance and increased adipocyte lipolysis plays a pivotal role in the development of this syndrome [1,8].

CIDE-A (cell-death-inducing DFF45-like effector-A) is a member of a family of proapoptotic proteins that includes CIDE-B and CIDE-3/FSP27 [9-11]. Whereas CIDE-A is capable of inducing apoptosis, CIDE-A also plays a role in regulating energy balance and lipid metabolism [12]. CIDE-A gene disrupted mice (CIDE-A -/-) have a lean phenotype and are resistant to diet-induced obesity and possibly diabetes [12]. CIDE-A also interacts and inhibits uncoupling protein-1 (UCP-1) resulting in greater energy expenditure in brown adipose tissue (BAT) and less lipid accumulation in white adipose tissue (WAT) [13]. Likewise, the lack of CIDE-A in gene disrupted mice results in increased thermogenesis, energy expenditure and lipolysis [14].

In humans, CIDE-A expression in adipose tissue is negatively correlated with fat mass [15,16]. That is, CIDE-A has been shown to be decreased 2-fold in subcutaneous WAT of obese humans yet highly upregulated in obese individuals undergoing weight reduction [16]. In addition, a single nucleotide polymorphism (V115F) has been shown to be associated with obesity in a Swedish population [17].

Previous reports have indicated that CIDE-A is not expressed in normal adult human or mouse liver tissue [9,12]. However, CIDE-A has been detected in the liver of mice treated with the hypolipidemic compound and potent peroxisome proliferator, WY-14,643 [18,19]. Due to the recent reports describing a role for CIDE-A in the regulation of lipid metabolism, we examined CIDE-A expression in liver of normal mice at various ages and in a mouse model of diet-induced type 2 diabetes and liver steatosis.

## Results

### CIDE-A is expressed in the liver of old mice

**Figure 1:**
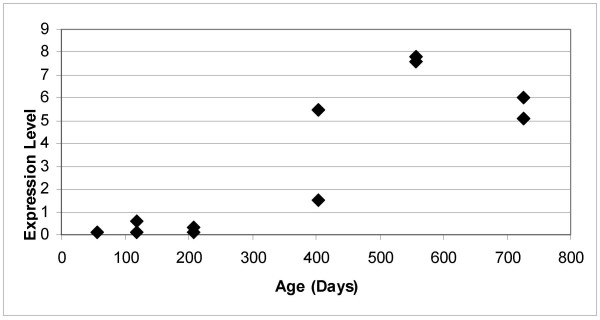
**CIDE-A is expressed in liver of aging mice**. Amersham CodeLink Expression Bioarrays™ were performed on biotinylated cRNA generated from poly(A) mRNA isolated from the liver of mice ranging from 56 to 725 days of age. Expression levels relate to fluorescence detected from processed DNA microarrays. The CIDE-A expression value in each individual liver is shown.

Microarray analysis was used to identify differences in liver gene expression in aging mice. The mice were sacrificed at ages ranging from 56 to 725 days. A total of 190 genes were differentially expressed by at least a 2-fold magnitude between 2 time points. Analysis identified CIDE-A as the most differentially expressed gene in liver during this age span (Fig. 1). CIDE-A expression was not detected at 56 days of age (expression level less than 0.2). The expression of CIDE-A was barely detectable at 118 and 207 days of age (0.59, 0.13 and 0.13, 0.34, respectively). However, CIDE-A is readily detected at 403 days of age (5.5, 1.5) and the level of expression continues to increase at 558 days of age (7.83, 7.59). Taken together, the level of CIDE-A expression in liver increases at least 38-fold as the mouse progresses from 56 days of age to maximal expression at 558 days of age.

### Liver steatosis is observed in CIDE-A expressing older mice

**Figure 2:**
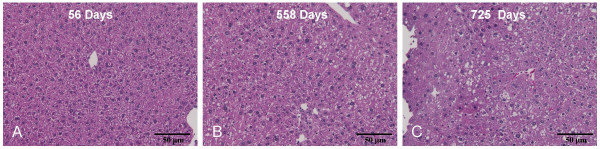
**Increased liver steatosis in older mice**. H&E stained liver sections isolated from mice fed standard chow at 56 (A), 558 (B) and 725 (C) days of age shows the accumulation of lipid in liver hepatocytes of older mice.

H&E stained liver sections prepared from mice of various ages were examined to determine if increased CIDE-A expression correlated with any noticeable histological changes in the livers of these mice (Fig. 2). Although only a single liver sample was analyzed at each time point, there was a tendency for the percent white space to increase with age (2 months = 7.98% *vs *18 months = 9.15% *vs *24 months = 9.98%). (While this observation is by no means conclusive, it does provide a basis for additional investigation.)

### CIDE-A expression is increased in type 2 diabetic mice

**Figure 3:**
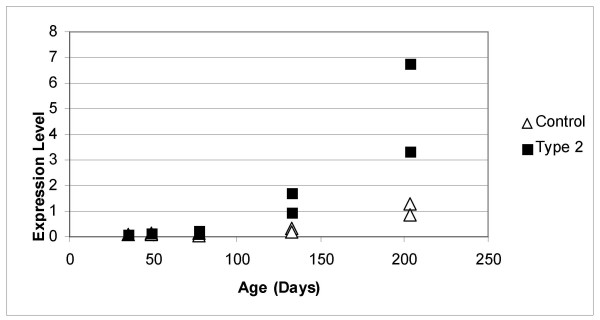
**CIDE-A expression is increased in the type 2 diabetic liver**. Amersham CodeLink Expression Bioarrays™ were performed on biotinylated cRNA generated from poly(A) mRNA isolated from the liver of mice ranging from 35 to 203 days of age (2, 4, 8, 16 and 26 weeks on diet). The CIDE-A expression value in each individual liver is shown. Black boxes: Type 2 diabetic mice fed the high fat diet. Open triangles: control mice fed standard chow. Expression levels relate to fluorescence detected from processed DNA microarrays.

Due to the correlation of increased CIDE-A expression with increasing age, we investigated whether CIDE-A expression would also be increased in a model of diet-induced obesity and type 2 diabetes [20-22]. DNA microarray analysis of RNA isolated from liver tissue of control and type 2 diabetic mice identified 466 genes whose expression is altered by at least 2-fold between normal and type 2 diabetic tissues. The level of CIDE-A expression in these tissues is shown in Figure 3.

**Figure 4:**
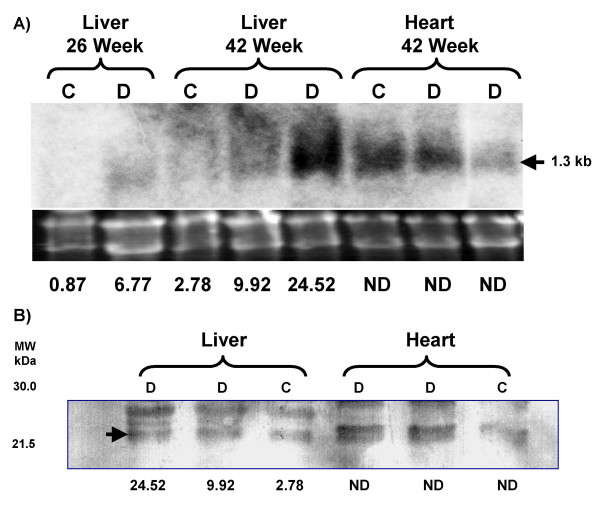
**CIDE-A Northern and Immunoblot analyses**. **A) **Northern analysis of RNA extracted from normal (C) and type 2 diabetic (D) liver and heart tissue. Total RNA (10 μg) from the appropriate tissues was resolved by denaturing agarose gel electrophoresis, transferred to positively charged nylon membrane, hybridized with the [α-^32^P]dCTP-labeled mouse CIDE-A cDNA and exposed to Bio-Max MR film. Ethidium bromide stain of RNA (10 μg/lane) prior to transfer to nylon membrane. The values represent the level of CIDE-A gene expression for the individual tissue as determined by DNA microarray analysis (ND: not determined). The approximated size (1.3 kb) of the CIDE-A mRNA is noted on the right. **B) **Immunoblot demonstrating increased CIDE-A protein levels in *type 2 *diabetic mouse liver. Sixty μg of liver and heart extract was electrophoresed on a 12.5% SDS-polyacrylamide gel and the resolved proteins transferred to a nitrocellulose membrane. The membrane was immunoblotted using a rabbit anti-mouse CIDE-A polyclonal antibody and a goat anti-rabbit IgG polyclonal antibody conjugated to horseradish peroxidase. Arrow indicates mouse CIDE-A. The values represent the level of CIDE-A gene expression for the individual tissue as determined by DNA microarray analysis (ND: not determined).

In agreement with the above data, CIDE-A expression (< 0.2 background) was not detected in the livers of mice fed the normal diet at 35, 49 or 77 days of age (2, 4, or 8 weeks on diet). The expression of CIDE-A was barely detectable at 133 days of age (16 weeks on diet: 0.31, 0.19) and begun to rise at 203 days of age (26 weeks on diet: 1.28, 0.87). In contrast, the expression of CIDE-A in the liver of type 2 diabetic mice fed a high-fat diet was detected at 77 days of age (8 weeks on diet: 0.25, 0.16) and continued to rise rapidly at 133 and 203 days of age (16 and 26 weeks on diet: 1.73, 0.96 and 3.34, 6.77, respectively). This represents a 5-fold increase in CIDE-A gene expression. We also performed Northern and immunoblot analyses of liver tissue to confirm the CIDE-A expression patterns (Fig. 4). The 1.3 kb CIDE-A mRNA was not detected in control liver samples whose CIDE-A microarray expression levels were 0.87 and 2.78. However, CIDE-A mRNA was faintly visible in the 26 week diabetic liver and was much more prevalent in the 42 week diabetic liver expressing high levels of CIDE-A. The CIDE-A expression levels as determined by microarray analysis were 6.77 and 24.52. CIDE-A mRNA was also detected in control and diabetic heart tissue as previously reported (9). Immunoblot analysis only detected CIDE-A in liver expressing the highest level of mRNA (42 week diabetic liver).

### Liver steatosis induced by high-fat diet

**Figure 5:**
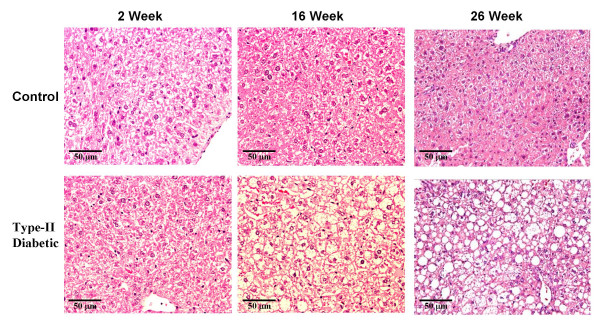
**Steatosis in liver of high-fat diet fed mice**. Mice were weaned directly onto either standard chow or a high-fat diet and maintained on the respective diets for up to 26 weeks. The mice were sacrificed and liver tissue isolated. Histology was performed on H&E stained liver tissue as described.

**Figure 6:**
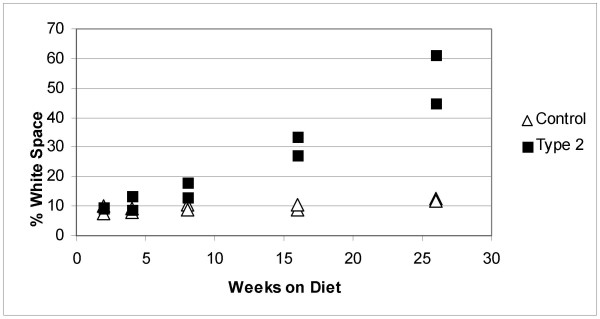
**Quantification of percent liver white space**. Image analysis was made as described in methods. The CIDE-A expression value in each individual liver is shown. Black boxes: Type 2 diabetic mice fed the high fat diet. Open triangles: control mice fed standard chow. Expression levels relate to fluorescence detected from processed DNA microarrays.

In addition to causing obesity and type 2 diabetes, the feeding of a high fat diet such as that used in these experiments to C57BL/6J mice, also causes lipid accumulation within the liver (steatosis) [23-28]. We performed histological examinations on H&E stained liver sections prepared from control and type 2 diabetic mice after 2, 16 and 26 weeks of feeding to assess the degree of liver steatosis (Figs. 5, 6).

Hepatocytes from control and diabetic mice contained approximately the same level of lipid at 2 (individual values – control: 10.2%, 7.7%; diabetic: 9.7%, 9.2%) and 4 weeks (individual values – control: 9.3%, 8.1%; diabetic: 13.6%, 8.8%) on the respective diets. However, by 8 weeks, hepatocytes from diabetic mice liver tissue isolated from high fat-fed mice contain more lipid than their control counterparts (individual values – control: 10.3%, 8.9%; diabetic: 18.0%, 13.0%). Severe liver steatosis was observed in mice fed the high-fat diet for 16 weeks and was even more pronounced after 26 weeks of high-fat feeding. The percent white space in these livers was 33.6% and 27.0% at 16 weeks and 45.0% and 61.3% at 26 weeks. In comparison, the percent white space in liver tissue of mice fed the normal diet for 16 weeks was 8.6% and 10.6% and is 12.5% and 11.8% for those at 26 weeks. The changes in percent white space were positively correlated with CIDE-A expression levels as determined by microarray analysis (*r *= 0.94; *P *< 0.001).

### Effect of diet-reversal on CIDE-A expression

**Figure 7:**
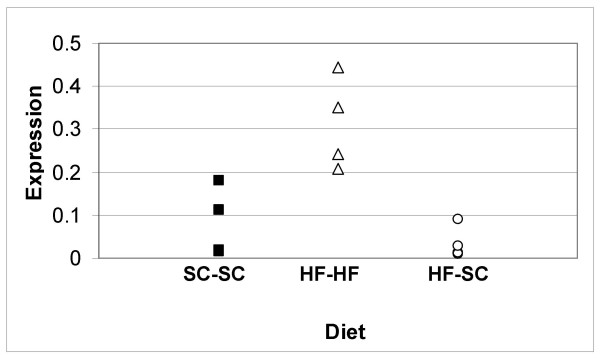
**Effect of diet-reversal on CIDE-A expression**. Mice were weaned onto the indicated diet for 33 weeks. The mice were then switched to the indicated diets for an additional 14 weeks. RNA was isolated from frozen liver as described. Real Time RT-PCR was performed on the cDNA transcripts using CIDE-A forward and reverse primers. Housekeeping genes, ACTG (actin gamma cytoplasmic) and GADPH (glyceraldehyde-III phosphate dehydrogenase), were utilized for normalization as described. Relative expression levels were calculated using normalization factors derived from geNorm analysis of ACTG and GADPH using the delta-delta CT method. CIDE-A expression levels in control mice fed standard chow for the entire feeding period (SC-SC) were given a value of 1.0. Changes in CIDE-A expression due to diet were expressed relative to the control mice. N = 4 (for each group). SC, standard chow; HF, high-fat diet.

Previous reports have indicated that the type 2 diabetic condition generated in high fat-fed mice can be reversed by returning type 2 diabetic mice to standard chow [29]. We therefore performed a similar diet-reversal study to determine its effects on CIDE-A expression in the liver. Animal weights, plasma insulin and fasting blood glucose levels at time of sacrifice are shown in Table I. Liver tissue was isolated from control, high fat-fed and diet reversed mice and CIDE-A expression levels were determined by real-time PCR (Fig. 7). In agreement with the above data, mice fed a high fat diet for the entire period (HF-HF) exhibited a statistically significant 3.8-fold increase in CIDE-A expression (0.21, 0.45, 0.24 and 0.30 vs 0.11, 0.18, 0.02 and 0.01 for HF and control, respectively). These mice also exhibited elevated weight and insulin levels relative to control mice. Mice switched to standard chow from a high fat diet (HF-SC) demonstrated normalized weight and insulin levels and a significantly lower blood glucose level. CIDE-A expression in these mice was also drastically reduced, falling to a level only 44% that seen in the control mice, although this decrease was not statistically significant (0.03, 0.09, 0.01 and 0.01 vs. 0.11, 0.18, 0.02 and 0.01 for diet-reversed and control, respectively). CIDE-A expression levels were positively correlated with both weight (*r *= 0.8; *P *< 0.005) and with insulin levels (*r *= 0.8; *P *< 0.005) but was not correlated with glucose levels. (These data indicate that CIDE-A expression was more closely correlated to plasma insulin levels and weight than to fasting blood glucose and suggests that increased CIDE-A expression precedes elevated blood glucose during the onset of obesity-induced type 2 diabetes.)

**Table 1:** Effect of diet reversal on weight, insulin and glucose levels of type 2 diabetic mice.

**Diet**	**Weight **(g)	**Plasma Insulin **(ng/ml)	**Fasting Glucose **(mg/dl)
SC-SC	**35.1 (3.5)**^a^	**326 (190)**^a^	**150 (13.8)**^a^
HF-HF	**56.5 (5.3)**^b^	**1981 (1170)**^b^	**153 (17.9)**^a^
HF-SC	**37.1 (1.9)**^a^	**247 (104)**^a^	**114 (11.4)**^b^

Values are: mean (SD). N = 4 (for each group). Means in a column without a common letter differ significantly, *P *< 0.05. SC denotes mice fed standard chow; HF denotes mice fed the high fat diet; HF-SC denotes diet-reversed mice that were first fed the high fat diet and switched to standard chow.

## Discussion

While liver steatosis and its associated diseases represent an ever increasing health problem, the key pathways and metabolic processes involved in the development of this disease are not fully understood and effective therapies are lacking. In this report, we describe a new pathway that may be involved in liver steatosis. We demonstrate that CIDE-A is expressed in liver of old mice. In fact, DNA microarray analysis indicates that CIDE-A is the most differentially expressed liver gene between young and older mice (38-fold increase). The increased expression of CIDE-A may not be due solely to increased age per se, but more likely a consequence of increased insulin resistance in the mice at older ages [30]. Insulin resistance is a common occurrence in aging individuals and is believed to be caused by increased adiposity rather than the aging process [31-36]. CIDE-A expression is also increased in a model of diet-induced type 2 diabetes. Increased CIDE-A expression was confirmed by Northern and immunoblot analyses. Elevated CIDE-A expression can be reversed by weight loss and normalization of plasma insulin. Also, CIDE-A expression was found to be correlated with hepatic lipid accumulation.

Previous reports have indicated that human and mouse CIDE-A are expressed in several tissues such as BAT, WAT, heart, lymph node, thymus, skeletal muscle and is localized to the mitochondria [9,15]. In another study, CIDE-A-deficient mice were found to have a lean phenotype and are resistant to obesity [12]. We believe that CIDE-A expression was not previously detected in liver due to the use of tissue from an inappropriate age or condition. Our data would suggest that human liver from older, insulin resistant or diabetic individuals may express this protein.

This study has identified CIDE-A as another potential mediator of lipid accumulation in liver hepatocytes. A recent study has proposed a human-specific role for CIDE-A in lipolysis and metabolic complications [15]. The previous study demonstrated that CIDE-A is expressed in human WAT with its expression decreased twofold in obese humans and normalized after weight loss. Reduced CIDE-A expression results in increased TNF-α secretion and basal lipolysis in subcutaneous WAT [15]. Increased TNF-α secretion also further decreases CIDE-A expression via TNF-α signaling through c-Jun NH_2_-terminal kinase (JNK) [15]. With this data, a model has emerged describing the role of CIDE-A in elevation of circulating FFA. Specifically, a decrease in CIDE-A expression results in increased TNF-α secretion resulting in increased lipolysis. The increased basal WAT lipolysis induced by low CIDE-A levels and elevated TNF-α secretion results in elevated levels of circulating fatty acid which can then be redirected to other tissues such as the liver. According to the model, increased CIDE-A expression and subsequent decreased TNF-α secretion would lead to decreased lipolysis and the accumulation of lipid within the tissue. Thus, increased CIDE-A expression in hepatocytes as a result of insulin resistance and type 2 diabetes may promote the uptake of increased circulating fatty acids released from WAT and lead to steatosis.

CIDE-A has been postulated to play a role in apoptosis suggesting the intriguing possibility that CIDE-A may play a role in apoptosis associated with liver steatosis and NAFLD. Disease progression from benign steatosis involves injury and an inflammatory response [4]. While the cause of the injury is not understood, it is clear that cellular apoptosis represents one of the first responses to injury and is a prominent feature of NAFLD as well as other diseases such as viral hepatitis, alcohol-induced liver disease, cholestatic liver diseases and ischemia/perfusion injury [3-7,37,38]. We did not, however detect increased apoptosis in CIDE-A expressing livers samples by TUNEL analysis and therefore believe that CIDE-A main function in the liver involves lipid metabolism.

## Conclusion

CIDE-A is expressed in normal adult mouse liver at older ages and is expressed in the liver of hyperinsulinemic and type 2 diabetic mice. CIDE-A expression positively correlates with liver steatosis in a mouse model of obesity-induced type 2 diabetes. These observations suggest a novel role for CIDE-A in the lipid accumulation characteristic of liver steatosis.

Future experiments that further delineate CIDE-A expression and effects will shed light on its function in the liver. Definitive experiments include placing the CIDE-A null mice, described by Zhou et al. [12], on the described high fat diet and assessing the effect of the lack of CIDE-A on liver steatosis and type 2 diabetes. The complementary experiment of generating transgenic mice expressing CIDE-A in the liver and assessing its effect under the same conditions may provide clues to CIDE-A function. Regardless, these data provide compelling evidence that CIDE-A exists in the liver and further suggests that CIDE-A expression levels are radically altered in mice as a function of age and/or metabolic state.

## Materials and methods

### Animal models used to identify differentially expressed liver genes

All experimental protocols were approved by the Ohio University Animal Care and Utilization Committee.

For the analysis of gene expression during the aging process, twelve male C57Bl/6J mice were fed standard chow (PMI Nutrition International Inc., Brentwood, MO, Prolab RMH3000) immediately following weaning. Mice were sacrificed at 35, 49, 56, 77, 133, 207, 403, 558 and 725 days of age. A portion of the left lateral lobe of the liver was placed in 4% paraformaldehyde and the remaining tissue was frozen in liquid nitrogen until processed for RNA isolation. For the analysis of normal versus diabetic liver gene expression, we utilized a mouse model of obesity-induced type 2 diabetes first described by Surwit [20] and replicated successfully in our laboratory and others [21,22]. Mice were weaned at 3 weeks onto either standard chow or a high-fat diet (BioServe, Frenchtown, NJ. #F1850) for up to 26 weeks. Representative mice were sacrificed after 2, 4, 8, 16 and 26 weeks on the diet (35, 49, 77, 133 and 203 days of age) and liver tissue was isolated. Mice fed standard chow served as control animals. Type 2 diabetic mice were characterized as having > 200 mg/dl fasting blood glucose and at least a 2-fold increase in fasting plasma insulin compared to control mice.

For diet-reversal analysis, C57Bl/6J mice were weaned onto a standard chow (SC) diet and maintained for 47 weeks. A second set of mice were weaned onto a high-fat diet (HF) diet for 33 weeks. One half of the high-fat fed mice were maintained on the high-fat diet while the other half was switched to standard chow for a period of 14 weeks. Food and water were supplied *ad libitum *throughout the course of the studies. Following the 47 week feeding period, the animals were sacrificed and liver tissue isolated as described above.

### Glucose and insulin levels

Blood-glucose levels were measured from blood taken from the tip of the tail of fasted (8 hr) mice using a One Touch glucometer (Life scan). All measurements occurred between 2:00 and 5:00 pm. Insulin concentrations were determined using the Ultrasensitive Rat Insulin ELISA kit (ALPCO, Windham, NH) as instructed by the manufacturer. Values were adjusted by a factor of 1.23 as determined by the manufacturer to correct for the species difference in cross-reactivity with the antibody.

### Microarray analysis

Total RNA was isolated from whole liver using the RNA STAT-60 Total RNA/mRNA Isolation Reagent according to the manufacturer's instructions (Tel-Test, Friendswood, TX). Biotinylated cRNA hybridization target was prepared by a linear amplification method as described in the manufacturer's instructions for CodeLink Expression Bioarrays™ (Amersham Biosciences). The oligonucleotide probes were provided by the Codelink Uniset Mouse I Bioarray (Amersham, product code 300013). CodeLink Expression Bioarrays™ contain 10,000 oligonucleotide probes, each specific to a well-characterized mouse gene. Using the cRNA target, the hybridization reaction mixture containing the biotinylated target cRNA was loaded into array chambers for bioarray processing as described in the manufacturer's instructions for CodeLink Gene Expression BioarraysTM (Amersham Biosciences). Each sample was hybridized at 37°C to an individual microarray. Hybridization was detected with an avidinated fluorescent reagent, Streptavidin-Alexa Fluor ^® ^647 (Amersham). Processed arrays were scanned using a GenePix 4000B Microarray Scanner (Axon Instruments, Inc.). Array images were acquired using the Amersham CodeLink Analysis Software (Release 2.2). A significant difference in expression between samples was defined as a minimum of 2-fold change in expression values.

### Real-time RT-PCR

RNA was isolated from frozen liver as described above. cDNA transcripts were created using iScript cDNA Synthesis Kit (Cat# 170-8891, Bio-Rad Laboratories, Hercules, CA). Real Time RT-PCR was performed on the Bio-Rad iCycler iQ Real Time PCR Detection System (Cat# 170-8740, Bio-Rad Laboratories) using IQ SYBR Green Supermix (Cat# 170-8882). CIDE-A primers (Forward Primer: 5'CTCGGCTGTCTCAATGTCAA3'; Reverse Primer: 5'CCGCATAGACCAGGAACTGT3' were designed using Primer3 online software [39]. Housekeeping genes, ACTG (actin gamma cytoplasmic) and GADPH (glyceraldehyde-III phosphate dehydrogenase) were utilized for normalization as described by Vandesompele et al. [40,39]. Relative expression levels were then calculated using normalization factors derived from geNorm analysis of ACTG and GADPH using the delta-delta CT method [40].

### Liver histology and image analysis

Liver tissues fixed in 4% paraformaldehyde were embedded in Tissue Path (Fisher Scientific, Pittsburgh, PA). Embedded tissue was sectioned for seven microns thickness, stained with H&E and evaluated by an automated light microscopy system, consisting of a 20 × lens with 0.5 NA, scanning stage, 3-color CCD camera and image analysis software and database (Icoria Inc, Research Triangle Park, NC). Images were taken at 0.64 micron resolution and were automatically assembled into montages. From 300 to 500 single images were captured to represent a single tissue section. These montages were then used for subsequent automated analysis of both gross anatomical features and fine tissue structures using automated pathology software [41,42]. Tissue metrics included counts, area, density and size of hepatocyte nuclei, non-hepatocyte nuclei, percent intracellular and extracellular white space. The feature intracellular white space calculated the amount of white space within hepatic boundaries, the appearance of which is consistent with lipid accumulation in this study. Application of these methods resulted in distinct tissue profiles for all animals.

### Northern analysis

Total RNA (10 μg) from appropriate tissues was resolved by denaturing agarose gel electrophoresis, transferred to nylon membrane, hybridized with the [α-^32^P]dCTP-labeled mouse CIDE-A cDNA (Random Primed DNA Labeling Kit, Roche, Indianapolis, IN) and exposed to Bio-Max MR film (Eastman Kodak Co., Rochester, NY).

### Immunoblot analysis

Liver and heart tissue (100 mg) was homogenized in 0.5 ml phosphate buffered saline containing 7.5 μl protease inhibitor cocktail (Sigma #P8340, St. Louis, MO). The samples were centrifuged for 5 min at 10,000 *g*. The supernatant was collected and protein concentration determined (Bio-Rad Laboratories #500-0006, Hercules, CA). Sixty μg of each extract was electrophoresed on a 12.5% SDS-polyacrylamide gel as described [43]. Resolved proteins were transferred to a nitrocellulose membrane and immunoblotted using a rabbit anti-mouse CIDE-A polyclonal antibody (QED Bioscience Inc., San Diego, CA) as previously described [43].

### Statistical analysis

Data are reported as dot plots or as mean (SD). Differences between two groups were assessed using the unpaired two-tailed Student's *t *test. For comparisons between multiple groups, ANOVA followed by Tukey's multiple-comparisons test was used. Analysis of correlations was done with Pearson correlation coefficients. Statistical significance was indicated by *P *value less than 0.05. SigmaStat statistics software (version 12.0; SPSS) was used for all calculations.

## Competing interests

The author(s) declare that they have no competing interests.

## Authors' contributions

BK coordinated the study, was responsible for animal selection, carried out the Northern blot analysis, immunoblot analysis and drafted the manuscript. KB, AK, SN ad MB performed the DNA microarray analysis and liver histology. DB and EL were responsible for animal model generation and statistical analysis. RC performed the Real-time RT-PCR analysis. JK conceived this study, participated in the study design and helped draft the manuscript. All authors have read and approved the content of the manuscript.
